# Bias among managers: Its prevalence across a decade and comparison across occupations

**DOI:** 10.3389/fpsyg.2022.1034712

**Published:** 2022-11-11

**Authors:** George B. Cunningham, Harper R. Cunningham

**Affiliations:** ^1^Laboratory for Diversity in Sport, Department of Sport Management, University of Florida, Gainesville, FL, United States; ^2^Emory University, Atlanta, GA, United States

**Keywords:** diversity and inclusion, bias, stereotypes, discrimination, management

## Abstract

Employees from minoritized and subjugated groups have poorer work experiences and fewer opportunities for advancement than do their peers. Biases among decision makers likely contributes to these patterns. The purposes of this study were to (a) examine the explicit biases and implicit biases among people in management occupations (e.g., chief executives, operations managers, advertising and promotions managers, financial managers, and distributions managers, among others) and (b) compare their biases with people in 22 other occupations. The authors analyzed responses from visitors to the Project Implicit website, including assessments of their racial, gender, disability, and sexual orientation biases from 2012 to 2021. Results indicate that managers expressed moderate levels of explicit and implicit bias across all dimensions. Managers differed from people in other occupations in roughly one-third of the comparisons. The biggest differences came in their implicit biases, with managers expressing more bias than people in other occupations. The study’s originality rests in the scope of the work (the authors analyzed data from over 5 million visitors representing 23 broad occupations); comparison of people in management occupations to those in other work settings; and empirically demonstrating the biases that managers have.

## Introduction

Though gains have been realized, racial minoritized people, women, people with disabilities, and people who are lesbian, gay, bisexual, transgender, and queer (LGBTQ+) continue to encounter poor experiences at work and limited opportunities for advancement, relative to their peers. One area of differences comes in representation among chief executives, where women and racial minoritized people are relatively unlikely to hold such roles (US Bureau of Labor Statistics, 2022a). There is also evidence of occupational segregation, as people with disabilities, when compared to workers without disabilities, are more likely to be in service positions than in management roles (US Bureau of Labor Statistics, 2022b). Beyond access to work, there are differences in the quality of people’s work experiences. Consider a recent study of US federal agencies, all of which were noted for their LGBTQ+ inclusiveness (Cech and Rothwell, 2020). The researchers found that LGBTQ+ employees reported poorer work experiences than their heterosexual and cisgender peers across 16 measures, including job satisfaction, fairness, and equitable work environments. The patterns of mistreatment were not evenly distributed though, as LGBTQ+ women and racial minoritized people had especially poor work outcomes. These findings align with considerable research showing differences in employment rates, pay, turnover, experiences with incivility, and other forms of mistreatment among people who differ from the typical majority members (Colella et al., 2017; Triana et al., 2019; Cech and Rothwell, 2020; Sabat et al., 2020).

One of the more common explanations for these patterns is bias among people making hiring decisions and managing employees (Riach, 2009; Foley and Williamson, 2018; Cunningham, 2019; Golik and Blanco, 2022; Hardy et al., 2022). For example, within the US, White, able-bodied, heterosexual men are frequently depicted as the norm for who a leader is and should be (Rosette et al., 2008; de Cristofaro et al., 2020; Fitzsimmons and Callan, 2020; Pellegrini et al., 2020; Salvati et al., 2021); thus, when choosing among potential candidates, decision makers might draw on these stereotypes to inform their decisions. Further, people from minoritized and subjugated groups commonly report mistreatment, confronting prejudices, and navigating (sometimes invisible) stigma associated with their identities (Jones et al., 2017; McCord et al., 2018; Johnson et al., 2020). This mistreatment can come from clients, coworkers, and managers. These biases can negatively impact the degree to which employees are able to present their authentic selves in the work environment, and their subjective and physical health, among other outcomes (Cunningham, 2015; Williams et al., 2019). Organizations with high levels of bias are likely to experience turnover among quality employees, suffer productivity losses, and encounter legal challenges, among other outcomes (McKay et al., 2007; Dhanani et al., 2018; Mao et al., 2019).

Scholarship focusing on bias in the workplace has shown the pernicious impact that stereotypes and prejudices can have on employees and the workplace overall. Nevertheless, importance gaps still remain. First, researchers have commonly examined employees’ experiences with bias (Jones et al., 2017; McCord et al., 2018) or conducted experimental studies to capture discriminatory behaviors, such as with resume correspondence audit studies (Quillian et al., 2017, 2019). Scholarship focusing on organizational decision makers is comparatively less common. To be sure, there are many conceptualizations of inclusive leadership (Nishii and Leroy, 2022; van Knippenberg and van Ginkel, 2022), and researchers have consistently shown that employers favor people in majority groups when reviewing resumes (Jost et al., 2009). These advancements noted, when compared to scholarship focusing on the targets of bias, empirical research focusing on biases expressed among the organizational decision makers is less common (Salles et al., 2019; Kershaw et al., 2021). Second, though some people express bias in explicit ways, much of the mistreatment in today’s workplace is more subtle in nature (Cortina et al., 2013; van Laar et al., 2019). Therein rests the importance of considering both explicit and implicit forms of bias among managers. Finally, if biased decision making among managers contributes to the poor work experiences of minoritized and subjugated groups, is there something unique about managers, or are their biases widely shared? The former would suggest that people in decision making roles are more likely to harbor biases than are those in other positions, but the latter would suggest that biases are common and other factors are more likely to blame. We address each of these areas in the current study. Specifically, in drawing from a large, publicly available database, we examine the implicit and explicit biases managers hold regarding race, women at work, disability, and members of the LGBTQ+ communities. We do so by analyzing ten years of data and compare managers’ responses to those from people working in other occupations.

## Theoretical framework

### Explicit and implicit forms of bias

From a social psychological perspective, stereotypes (i.e., the cognitive form of bias) and prejudice (i.e., the affective form of bias) can manifest explicitly or implicitly (Dovidio et al., 2010). Explicit biases are those people consciously hold and deliberately maintain. They can articulate their explicit biases to others, such as when responding to pollsters or completing questionnaires. For example, in a meta-analysis of field experimental studies, Ren et al. showed that hiring personnel had lower performance expectations for people with disabilities (Ren et al., 2008). The bias directed toward people with mental disabilities was especially strong. As another example, Burton et al. conducted an experimental study whereby athletic administrators in the US reviewed vignettes of job applicants and provided ratings along various dimensions (Burton et al., 2011). The administrators rated women applying for the athletic director position (the top post in these organizations) as less feminine than women applying for other leadership roles. Thus, ideas about who should lead a sport organization were closely aligned with masculinity and privileged men or masculine-presenting women. In these examples, and others like them, participants express their attitudes and beliefs on a questionnaire, sharing their explicitly held biases toward the targets.

In addition to explicit forms of bias, people express implicit bias, or their automatic, unintentional associations with different targets (Vuletich and Payne, 2019). Implicit biases manifest when there is a match between a target and the attributions people have toward that target (Dovidio et al., 2002). Though implicit biases are automatically activated, people are aware of them and can predict their occurrence with reasonable accuracy (Hahn et al., 2014). In addition, implicit biases frequently arise when people are faced with equivocal information or when social cues to respond in a particular way are weak (Son Hing et al., 2008). That is, implicit biases are unlikely to manifest when the solution is clear or when (for example) one applicant is demonstratively more qualified than another. Instead, implicit biases arise when the correct decision is debatable or when the clear path forward is unknown—the very situations in which managers or hiring personnel frequently find themselves (Derous et al., 2015).

Researchers have shown that peoples’ implicit biases can affects their beliefs and behaviors. For example, people who have implicit racial bias are also likely to endorse racial stereotypes about athletic performance of minoritized people (Furley and Dicks, 2014). Similarly, poor services provided by healthcare providers are frequently driven by their implicit biases toward the patient (Maina et al., 2018). Implicit biases also appear on committees, not just among individuals. Researchers have shown that committees that express collective gender bias are less likely to support women for promotions (Régner et al., 2019). In the organizational context, Zaniboni et al. showed that implicit biases among managers corresponded with more negative evaluations of older job applicants (Zaniboni et al., 2019). In another study, Rooth showed that for every one standard deviation increase in implicit bias, the likelihood of hiring an Arab-Muslim job applicant decreased by five points (Rooth, 2010).

Though they are both measures of bias, explicit and implicit biases do not always correspond (Dovidio et al., 2010). People can think of themselves as fair-minded or as people who hold egalitarian views, but even when holding those perspective, they still express implicit bias. Two examples help illustrate. Friedman conducted a large-scale study of people who had a family member with a disability (Friedman, 2019). When asked to respond to explicit measures, the family members did not express a bias against people with disabilities; however, they did hold implicit biases against such individuals. Likewise, Cunningham and Melton conducted a qualitative study with parents who had previously expressed support for coaches who identified as lesbian, gay, and bisexual (i.e., explicit support) (Cunningham and Melton, 2014). Even though the parents had earlier indicated they supported the coaches, about half still held biases that served to reinforce harmful, antiquated stereotypes about people from the LGBTQ+ community. The authors attributed these beliefs to the participants’ implicit biases, but they did not empirically assess as much.

### Current study

The purpose of the current study was to examine the implicit and explicit biases of managers working in the US, where managers are people charged with securing and allocating resources (i.e., human, financial, physical, and informational resources) to achieve organizational objectives (Griffin, 2016). Examples include chief executives, operations managers, advertising and promotions managers, financial managers, and distributions managers, among others. As outlined in more detail in the following section, we did so by drawing from the Project Implicit database—a publicly available repository with implicit bias data from millions of people. Recognizing that biases exist across a wide range of domains, we limited our analyses to four areas: race, gender, disability, and sexual orientation. We did so for several reasons. First, according to the Equal Employment Opportunity Commission (EEOC), charges based on disability, race, and gender are among the most prevalent forms of bias and mistreatment in US workplaces (Equal Employment Opportunity Commission, 2021). Thus, among people’s characteristics protected by employment laws, race-, gender- and disability-based mistreatment are most prevalent. At the time of the data collection, people did not receive employment protections based on their sexual orientation; thus, EEOC data were not available. However, reports show that roughly 45% of American workers who identify as LGBTQ+ report encounters with workplace discrimination during their careers (Sears et al., 2021). Thus, race-, gender-, disability-, and sexual orientation-based forms of mistreatment are all prevalent in the US workforce, warranting examination of managers’ biases in these areas.

Based on the literature and theory reviewed, we developed the following research questions:

RQ1: What is the level of explicit bias (RQ1a) and implicit bias (RQ1b) among managers in the US by type: race, gender, disability, and sexual orientation?

RQ2: Do expliciti biases (RQ2a) and implicit biases (RQ2b) exhibited among managers in the US differ from the biases exhibited by people in other occupations?

## Materials and methods

### Data source

To examine our research questions, we analyzed publicly available data from Harvard’s Project Implicit.^1^ Project Implicit is a non-profit organization focused on educating people about prejudices, stereotypes, and other biases people hold. Visitors to the website can complete online assessments, which include an evaluation of their explicit and implicit biases toward different targets. Website visitors are also asked to complete demographic information, such as their race, gender, and occupation, among others. The Project Implicit team posts the anonymized data online at the Center for Open Science,^2^ an Internet site where researchers post data, papers, protocols, and other research materials, all with the aim of promoting open science.

Researchers have drawn from these data to investigate a variety of topics, including income inequality (Connor et al., 2019), biases across 34 countries around the world (Charlesworth et al., 2022), and the influence of racial bias on death rates (Leitner et al., 2016a), among others. In an example of how researchers have used the dataset to examine workplace issues, Cunningham and Nite investigated how sexual orientation implicit biases at the state level were related to the diversity and inclusion efforts among sport organizations in that state, as well as the organization’s effectiveness (Cunningham and Nite, 2020). Given the widespread use of the data and its relevance in answering our research questions, we drew from the Project Implicit datasets in the current study.

### Variables

We analyzed implicit and explicit bias data related to race, gender-career, disability, and sexual orientation, as well as the occupation of the respondents, and the racial and gender composition of the people in that occupation.

**Implicit bias.** Implicit biases were assessed using the Implicit Association Test, or IAT (Greenwald et al., 1998). As Greenwald et al. explain (Greenwald et al., 2009), the test “assesses strengths of associations between concepts by observing response latencies in computer-administered categorization tasks” (p. 18). As such, the IAT focuses on automatic associations rather than people’s deliberate responses. Researchers using the IAT calculate differences in response times between associations to determine the preference for one group (e.g., a person without a disability) relative to others (e.g., a person with a disability). The differences scores can theoretically range from –2 to +2, though most fall between –1 and +1. Researchers can then classify the level of preference or bias as neutral (0–0.15), slight (0.16–0.35), moderate (0.36–0.65), or strong (greater than 0.65) (Greenwald et al., 2003). In their meta-analysis, Greenwald et al. showed evidence of the IAT’s test-retest reliability, validity evidence, and internal consistency (Greenwald et al., 2009).

**Explicit bias.** Explicit attitudes related to race, disability, and sexual orientation were assessed using the Feeling Thermometer. Participants responded to two items regarding how warm or cold they felt. For example, for race, the items read, “please rate how warm or cold you feel toward White people” and “please rate how warm or cold you feel toward Black people.” Response options ranged from 0 (coldest feelings) to 10 (warmest feelings). Consistent with previous researchers using the Project Implicit dataset to explore explicit bias (Leitner et al., 2016b; Hehman et al., 2018; Cunningham and Wigfall, 2020), we subtracted ratings of the marginalized group from those of the majority group (e.g., Feeling Thermometer rating for White people—Feeling Thermometer rating for Black people). Thus, higher scores reflected a strong preference White people, able-bodied people, and heterosexual, respectively. For explicit attitudes related to gender, people responded to two items: “how strongly do you associate career with males and females” and “how strongly do you associate family with males and females.” Responses options for both questions were: 1 (*strongly female*), 2 (*moderately female*), 3 (*slightly female*), 4 (*neither male nor female*), 5 (*slightly male*), 6 (*moderately male*), and 7 (*strongly male*). We measured explicit bias by subtracting the family ratings from the work ratings. Higher scores reflected a stronger preference for men in the workplace.

**Occupation.** Participants provide their occupation when completing the surveys, and the Project Implicit dataset then has them grouped based on the US Bureau of Labor Statistics Standard Occupational Classification System.^3^ People working as managers are grouped under the broad Management Occupations umbrella, including executives; advertising, marketing, promotions, public relations, and sales managers; operations specialties managers, and other management occupations. We then compared the responses of people in management occupations to those from the other 22 occupations, as listed in Table 1.

**TABLE 1:** Occupations, number of respondents, and participant demographics.

Occupation	Respondents	Percent white people (%)	Percent women (%)
Management	360,890	78.7	52.0
Business and financial	470,795	74.3	58.0
Computer and mathematics	167,695	67.6	45.4
Architecture and engineering	172,818	71.2	32.3
Life, physical, and social science	142,473	77.0	55.2
Community and social service	196,049	77.8	67.8
Legal	147,899	76.6	61.5
Educational instruction and library	1,282,257	77.5	70.4
Arts, design, entertainment, sports, and media	249,758	73.3	57.5
Healthcare practitioners and technical	169,720	73.4	61.4
Healthcare support	386,986	75.1	72.7
Protective services	48,274	74.5	39.7
Food preparation and serving	415,284	74.6	62.3
Building and grounds cleaning and maintenance	24,029	76.9	30.2
Personal care and service	90,998	75.8	62.7
Sales	395,691	72.6	63.4
Office and administrative support	240,840	67.5	66.9
Farming, fishing, and forestry	30,202	83.0	43.0
Construction and extraction	35,940	76.0	33.7
Installation, maintenance, and repair	18,505	74.9	23.0
Production	40,569	71.9	47.6
Transportation and material moving	30,085	71.1	45.5
Military specific	69,445	69.4	42.4

**Occupational demographics.** The specific demographic information within each dataset varied, but common to each was the participants’ gender and race. Thus, as a way to contextualize the findings, we analyzed data about the percent of people within each occupation who identified as White and as a woman.

### Analyses

The Project Implicit data are available by focus of the bias (e.g., disability, race) and then grouped by year. We collected data for each bias category from 2012 to 2021, resulting in 40 datasets. We computed the mean explicit bias and implicit bias scores for each occupation, meaning that the unit of analysis moved from the 5,187,211 individual responses to 7,420 bias scores for 23 occupations.

We examined the first research questions by computing the mean explicit bias score and mean implicit bias score for each occupation. For the second research question, we first standardized the variables (Nosek et al., 2009) and then computed two weighted analyses of covariance, controlling for the year of the data collection, percent of White people in the occupation responding, and percent of women in the occupation responding. The amount of bias served as the dependent variable, and bias category and occupation served as the independent variables. Where significant differences were observed, we computed *post-hoc* tests of the estimated marginal means, using Sidak correction. With respect to the weights, given the differences in the number of responses in each occupation, we followed Nosek et al. and computed the log of the inverse weights based on the standard errors (Nosek et al., 2009). We then averaged the two weights, creating a single weight for the analyses. Doing so allowed for accurate estimates across occupations with varying number of responses.

## Results

### Sample

As seen in Table 1, the dataset included responses from 5,187,202 people over the 10 years. Most of the participants identified as White (74.92%, *n* = 3,885,829) and most as women (61.17%; *n* = 3,173,057). Twenty-three occupations were represented, with people working in Educational Instruction and Library the best represented and those in Installation, Maintenance, and Repair with the fewest respondents.

### Explicit and implicit biases of people in management occupations

With our first set of research questions, we examined the levels of explicit and implicit biases among people in management occupations. Mean explicit bias scores of zero represent a lack of bias (e.g., rating people with disabilities as positively as one rates people without disabilities), whereas a positive score represents a preference for the majority group, and a negative score represents a preference for the minoritized or subjugated group. As seen in Table 2, with respect to explicit biases, each of the mean scores was positive, indicating that people working in Management occupations had an explicit bias in favor of people without disabilities, men (relative to women) working outside the home, White people, and heterosexual people.

**TABLE 2:** Means and standard deviations of explicit bias and implicit bias, across occupations.

Occupation	Disability		Gender		Race		Sexual orientation	
	M	SD	M	SD	M	SD	M	SD
**Explicit bias**								
Management	0.140	0.196	1.322	0.340	0.114	0.259	0.621	0.366
Business and financial	0.196	0.180	1.360	0.381	0.232	0.288	0.922	0.453
Computer and mathematics	0.156	0.438	1.139	0.293	0.272	0.254	0.531	0.531
Architecture and engineering	0.197	0.394	1.163	0.338	0.315	0.315	0.916	0.539
Life, physical, and social science	0.053	0.231	1.112	0.255	0.074	0.301	–0.035	0.456
Community and social service	0.077	0.320	1.178	0.324	–0.146	0.263	0.240	0.730
Legal	0.040	0.220	1.240	0.314	–0.103	0.295	0.251	0.435
Educational instruction and library	0.030	0.157	1.114	0.305	–0.106	0.259	0.117	0.493
Arts, design, entertainment, sports, and media	0.114	0.418	0.971	0.255	–0.161	0.281	–0.116	0.461
Healthcare practitioners and technical	0.115	0.166	1.245	0.345	0.153	0.311	0.581	0.578
Healthcare support	–0.065	0.087	1.158	0.317	0.096	0.276	0.686	0.475
Protective services	0.077	0.419	1.185	0.462	0.282	0.434	1.205	0.594
Food preparation and serving	0.227	0.246	1.168	0.363	0.082	0.413	0.244	0.564
Building and grounds cleaning and maintenance	–0.020	0.352	1.040	0.435	0.363	0.479	1.596	0.644
Personal care and service	–0.023	0.446	1.082	0.496	0.160	0.526	0.242	0.763
Sales	0.165	0.266	1.271	0.364	0.109	0.412	0.601	0.481
Office and administrative support	0.030	0.183	1.263	0.328	–0.163	0.238	0.450	0.489
Farming, fishing, and forestry	0.168	1.123	1.048	0.456	0.931	0.693	1.554	1.604
Construction and extraction	0.224	0.557	1.183	0.381	0.503	0.376	1.836	1.065
Installation, maintenance, and repair	0.301	0.845	1.007	0.483	0.394	0.567	1.300	1.208
Production	0.191	0.660	1.183	0.466	0.216	0.398	0.969	0.542
Transportation and material moving	0.038	0.710	1.185	0.488	0.217	0.446	1.006	0.640
Military specific	0.229	0.240	1.327	0.406	0.278	0.397	1.218	0.507
**Implicit bias**								
Management	0.588	0.035	0.372	0.026	0.310	0.030	0.244	0.056
Business and financial	0.581	0.031	0.368	0.025	0.325	0.032	0.279	0.063
Computer and mathematics	0.503	0.114	0.299	0.041	0.315	0.045	0.202	0.081
Architecture and engineering	0.549	0.053	0.314	0.040	0.323	0.054	0.228	0.102
Life, physical, and social science	0.480	0.063	0.330	0.027	0.285	0.043	0.089	0.068
Community and social service	0.469	0.054	0.385	0.031	0.253	0.020	0.177	0.058
Legal	0.536	0.032	0.373	0.035	0.290	0.030	0.174	0.058
Educational instruction and library	0.488	0.044	0.382	0.033	0.268	0.035	0.135	0.081
Arts, design, entertainment, sports, and media	0.436	0.059	0.317	0.036	0.247	0.036	0.061	0.071
Healthcare practitioners and technical	0.542	0.030	0.369	0.033	0.326	0.030	0.214	0.071
Healthcare support	0.530	0.031	0.402	0.038	0.309	0.029	0.263	0.079
Protective services	0.601	0.124	0.335	0.052	0.327	0.049	0.369	0.100
Food preparation and serving	0.484	0.047	0.353	0.036	0.312	0.037	0.181	0.088
Building and grounds cleaning and maintenance	0.575	0.107	0.285	0.056	0.302	0.057	0.346	0.073
Personal care and service	0.494	0.094	0.376	0.079	0.314	0.070	0.174	0.125
Sales	0.537	0.054	0.371	0.032	0.311	0.037	0.255	0.080
Office and administrative support	0.534	0.049	0.396	0.031	0.274	0.027	0.240	0.069
Farming, fishing, and forestry	0.479	0.179	0.295	0.069	0.339	0.051	0.234	0.149
Construction and extraction	0.570	0.122	0.276	0.051	0.341	0.038	0.355	0.084
Installation, maintenance, and repair	0.599	0.083	0.275	0.065	0.335	0.048	0.375	0.110
Production	0.585	0.134	0.330	0.073	0.318	0.068	0.323	0.098
Transportation and material moving	0.595	0.104	0.344	0.069	0.316	0.065	0.305	0.096
Military specific	0.564	0.065	0.325	0.040	0.314	0.061	0.349	0.071

Values shown without weights.

For implicit bias scores, we use the benchmarks identified by Greenwald et al.: neutral (0–0.15), slight (0.16–0.35), moderate (0.36–0.65), or strong (greater than 0.65) (Greenwald et al., 2003). For each area of bias, people working in management occupations held a moderate preference for people without disabilities, men (relative to women) working outside the home, White people, and heterosexual people.

### Differences in explicit biases across occupations

Turning to the next set of research questions, we first computed the weighted analysis of covariance for explicit biases. Each of the three covariates (i.e., year, percent of White respondents, percent of women respondents) was significant. The main effects of bias category, *F* (3, 3,596) = 2,081.76, *p* < 0.001, and occupation, *F* (22, 3,596) = 38.34, *p* < 0.001, were both significant. The main effects were qualified, however, by a significant occupation × bias category interaction, *F* (21, 3,596) = 19.84, *p* < 0.001.

**Explicit disability bias.** *Post-hoc* analyses showed that people in management occupations did not differ from those in other occupations with respect to explicit disability bias.

**Explicit gender bias.** One difference materialized for explicit gender bias, as people in management occupations had greater bias than did those in arts, design, entertainment, sports, and media (*p* = 0.019).

**Explicit racial bias.** Several differences did emerge in explicit racial bias. People in management occupations expressed significantly more explicit racial bias than did those in social services (*p* = 0.036); education instruction and library (*p* = 0.004); arts, design, entertainment, sports, and media (*p* = 0.025); and office and administrative support (*p* = 0.005) occupations. On the other hand, people in management positions expressed significantly less explicit racial bias than did those in farming, fishing, and forestry (*p* < 0.001) and construction and extraction (*p* < 0.001) occupations.

**Explicit sexual orientation bias.** People in management occupations also varied from others in their explicit sexual orientation bias. Specifically, they expressed more bias than did people in life, physical, and social sciences (*p* < 0.001); community and social services (*p* = 0.048); education instruction and library (*p* < 0.001); arts, design, entertainment, sports, and media (*p* < 0.001); food preparation and serving (*p* = 0.003); and personal care and service (*p* < 0.001) occupations. However, people in management occupations also expressed less explicit sexual orientation bias than those in business and financial (*p* < 0.001); architecture and engineering (*p* = 0.007); building and grounds cleaning and maintenance (*p* < 0.001); farming, fishing, and forestry (*p* < 0.001); construction and extraction (*p* < 0.001); installation, maintenance repair (*p* < 0.001); production (*p* < 0.001); transportation and material moving (*p* < 0.001); and military service (*p* < 0.001) occupations.

### Differences in implicit biases across occupations

Turning to the second weighted analysis of covariance, year and the percent of White respondents from the occupation were significant covariates of implicit racial bias. The percent of women respondents in the occupation was not. The main effects of bias category, *F* (3, 3,596) = 3,868.34, *p* < 0.001, and occupation, *F* (22, 3,596) = 58.36, *p* < 0.001, were both significant. The main effects were qualified, however, by a significant occupation × bias category interaction, *F* (21, 3,596) = 26.81, *p* < 0.001. We therefore computed the *post-hoc* analyses.

**Implicit disability bias.** Results indicated that people in management occupations express more implicit bias against people with disabilities than do people in computer and mathematics (*p* = 0.023); life, physical, and social science (*p* < 0.001); community and social service (*p* < 0.001); educational instruction and library (*p* < 0.001); arts, design, entertainment, sports, and media (*p* < 0.001); food preparation and serving (*p* < 0.001); personal care and service (*p* < 0.001); sales (*p* = 0.004); office and administrative support (*p* = 0.018); and farming, fishing, and forestry (*p* < 0.001) occupations. They did not have less bias than any of the other occupations.

**Implicit gender bias.** With respect to gender, people in management occupations expressed more implicit bias than did people in business and financial (*p* < 0.001); architecture and engineering (*p* = 0.032); arts, design, entertainment, sports, and media (*p* < 0.001); building and grounds cleaning and maintenance (*p* < 0.001); farming, fishing, and forestry (*p* < 0.001); construction and extraction (*p* < 0.001); and installation, maintenance and repair (*p* < 0.001) occupations.

**Implicit racial bias.** Results showed only three differences in racial implicit bias. People in management occupations expressed more bias than did those in community and social service (*p* = 0.005); educational instruction and library (*p* = 0.008); arts, design, entertainment, sports, and media (*p* < 0.001) occupations.

**Implicit sexual orientation bias.** Finally, for sexual orientation implicit bias, people in management occupations expressed more bias than did those in life, physical, and social science (*p* < 0.001); community and social service (*p* = 0.006); legal (*p* = 0.003); educational instruction and library (*p* < 0.001); arts, design, entertainment, sports, and media (*p* < 0.001); food preparation and serving (*p* < 0.001); and personal care and service (*p* < 0.001) occupations. On the other hand, people in management occupations expressed less sexual orientation bias than did those in protective services (*p* < 0.001); building and grounds cleaning and maintenance (*p* < 0.001); construction and extraction (*p* < 0.001); installation, maintenance and repair (*p* < 0.001); production (*p* < 0.001); transportation (*p* < 0.001); and military specific (*p* < 0.001) occupations.

### Summary of main analyses

In Table 3, we offer a summary of the findings. Of the 176 comparisons, statistically significant differences emerged in 58, or about a third of the time. Of those, people in management occupations expressed more explicit bias 12 times (6.82%), less explicit 10 times (5.68%), more implicit bias 27 times (15.70%), and less implicit bias 7 times (5.51%).

**TABLE 3:** Summary of differences in explicit and implicit biases between people in management occupations and people in other occupations.

Occupation	Disability		Gender		Race		Sexual orientation	
	Explicit	Implicit	Explicit	Implicit	Explicit	Implicit	Explicit	Implicit
Business and financial							–	
Computer and mathematics		+		+				
Architecture and engineering				+			–	
Life, physical, and social science		+					+	+
Community and social service		+			+	+	+	+
Legal								+
Educational instruction and library		+			+	+	+	+
Arts, design, entertainment, sports, and media		+	+	+	+	+	+	+
Healthcare practitioners and technical								
Healthcare support		+						
Protective services							–	–
Food preparation and serving		+					+	+
Building and grounds cleaning and maintenance				+			–	–
Personal care and service		+					+	+
Sales		+						
Office and administrative support		+			+			
Farming, fishing, and forestry		+		+	–		–	
Construction and extraction				+	–		–	–
Installation, maintenance, and repair				+			–	–
Production							–	–
Transportation and material moving							–	–
Military specific							–	–

+ Denotes people working in management occupations express more bias; – denotes people working in management occupation express less bias.

### Supplemental analysis

In addition to examining the specific research questions, we also investigated whether there were significant differences in the biases among managers. We did so by again computing weighted analyses of covariance, including the log of the inverse weights based on the standard errors as the weighting variable and the year, percent of White respondents, and percent of women respondents as covariates. The standardized explicit bias and implicit bias scores served as the dependent variables, respectively, and the type of bias served as the independent variable. Finally, we limited the analyses to include only people working in management occupations.

For explicit biases, the results were significant, *F* (3, 130) = 590.30, *p* < 0.001 (see Table 4). Among the covariates, only the year of data collection was significant. Sidak’s *post-hoc* test showed that the standardized mean scores for explicit disability bias and explicit racial bias did not significantly differ, and both were significantly lower than explicit gender bias and explicit sexual orientation bias. Explicit gender biases were significantly higher than the other biases.

**TABLE 4:** Differences in explicit and implicit bias among managers.

Category	Explicit bias		Implicit bias	
	EMM	SE	EMM	SE
Disability	−0.612^a^	0.035	1.625^d^	0.041
Gender	0.954^c^	0.031	0.054^c^	0.037
Race	−0.608^a^	0.030	−0.372^b^	0.035
Sexual orientation	0.022^b^	0.038	−0.820^a^	0.044

Values represent estimated marginal means of standardized bias variables, controlling for the percent of White respondents, percent of women respondents, and year. Different superscripts within a column reflective of statistically significant estimated marginal mean values.

For implicit biases, the results were significant, *F* (3, 130) = 634.26, *p* < 0.001. As seen in Table 4, the Sidak *post-hoc* analyses indicated that the estimates marginal mean scores for each of the implicit bias categories significantly differed from one another. Whereas explicit disability biases were lowest, implicit disability biases were significantly higher than all other bias categories. Implicit gender biases were next highest, followed by implicit racial bias and implicit sexual orientation, respectively.

## Discussion

People from minoritized and subjugated groups have poorer work experiences and fewer opportunities for advancement than do their peers, and biased decision making among organizational leaders potentially contributes to these patterns (Riach, 2009; Foley and Williamson, 2018; Cunningham, 2019; Golik and Blanco, 2022; Hardy et al., 2022). The purposes of this study were to (a) examine the explicit biases and implicit biases among people in management occupations, and (b) compare their biases with people in 22 other occupations. Results showed that people in management occupations expressed both explicit and implicit biases based on disability, gender, race, and sexual orientation. Thus, part of the reason for the consistent patterns of employee mistreatment (Jones et al., 2017; McCord et al., 2018) could be due to the explicit and implicit biases expressed by managers. People in management occupations also differed from approximately a third of the comparison groups. Finally, there was incongruence in some of the managers’ implicit and explicit biases; for example, whereas they expressed lower levels of explicit disability bias, their implicit disability biases were the highest of the four categories examined. In the following space, we note contributions of the research, identify limitations, and point to future directions.

### Contributions and implications

Stereotypes and prejudices harm the workplace experiences and advancement opportunities for people from minoritized and subjugated backgrounds. Further, data from the EEOC, coupled with large-scale studies on the topic (Equal Employment Opportunity Commission, 2021; Sears et al., 2021), show that mistreatment of people based on their disability, gender, race, and sexual orientation are among the most common in US workplaces. Despite these patterns, investigations of biases among people working in management occupations—that is, the very people overseeing the workplace, facilitating the hiring, and deciding on promotions—has been lacking. Thus, one contribution of the current study is remedying this shortcoming, as our analyses included responses from over 360,000 managers (and over 5 million total people).

Second, we showed a disconnect between managers’ explicit and implicit bias ratings, especially when it came to disability. These findings are consistent with recent disability research, such as the aforementioned study of people with a family member who had a disability (Friedman, 2019). Other scholarship in this area has largely focused on people working in healthcare (VanPuymbrouck et al., 2020; Lum et al., 2021; Feldner et al., 2022). Though people working in these fields consistently report little to no explicit bias, they express implicit preferences for people without a disability—patterns that have prompted use of the term ‘implicit ableists’ (VanPuymbrouck et al., 2020; Feldner et al., 2022). The data in this study point to a similar pattern among people working in management occupations within the US, suggesting that many in this work areas are also implicit ableists. Thus, the poor experiences and limited access for people with disabilities (Bjørnshagen and Ugreninov, 2021; Lyubykh et al., 2021) could be due, in part, to the implicit biases managers have against them.

People working in management occupations also differed from approximately a third of the comparison groups. As seen in Table 3, managers were most like people working as healthcare practitioners (e.g., medical doctors) and those in the business and financial sector (e.g., business operations specialists). People working in management occupations also expressed less bias (particularly related to sexual orientation) than those working in production (e.g., food process workers), transportation and material moving (e.g., railway workers), protective services (e.g., fire fighters), and those in the military. Finally, people in management occupations expressed more bias than people in community and social services (e.g., counselors and social workers), educational instruction and library (e.g., schoolteachers), arts, design entertainment, sports, and media (e.g., artists), and to a lesser degree, those working in life, physical, and social science (e.g., conservation scientists), food preparation and serving, and personal care and service (e.g., entertainment workers). These patterns suggest that managers have similar biases to people working in professional and white-collar occupations, less bias than those working in physical labor and blue-collar occupations, and more bias than people whose work involves bettering the human condition and protecting the environment.

The pattern of findings indicates that people in a given occupation, or even collective of occupations (as with managers and others in professional and white-collar occupations) might have shared understandings and assumptions (Zhou, 2005). From an institutional theory perspective, these understandings and assumptions are socially constructed; result from societal values, beliefs, rules, and so on; and help provide meaning to people’s social realities (Thornton and Ocasio, 1999). If this is the case, then people in management occupations might develop shared biases about people. Of course, individuals within the broader occupation might not share the biases, but the occupation as a collective does. These ideas are compatible with those from Payne et al., who suggested that biases might be best understood not from an individual level but from a community perspective, or the bias of crowds (Payne et al., 2017). Future researchers should explore the degree to which people in different occupations share common biases toward people and the reasons for these beliefs.

Finally, we also observed differences in the focus of the bias. People in management occupations were more likely to differ from others in their implicit disability biases and, to a lesser degree, their sexual orientation biases. Thus, even though they might not state as much explicitly, people in management occupations are more likely than their peers to express bias against people with disabilities and against members of the LGBTQ+ community. Put another way, the well-documented poor treatment of and limited advancement opportunities for people with disabilities (Bjørnshagen and Ugreninov, 2021; Lyubykh et al., 2021) and members of the LGBTQ+ community (Webster et al., 2018; Mara et al., 2021) are at least partially due to the biases held by managers.

### Implications, limitations, and future directions

These findings point to implications for practice, too, including the continued need for bias reduction strategies. Several options exist, as Paluck et al. noted in their review of the literature (Paluck et al., 2021). Most of these interventions focus on explicit attitudes, but studies of implicit bias reduction have demonstrated moderate effects. That noted, much of the scholarship in this area has employed what Paluck et al. refer to as light touch interventions, which are unlikely to have lasting effects. Instead, continued prejudice reduction efforts, in psychologically safe environments, that also foster connections with the organization are likely to be most effective (Creon and Schermuly, 2019; Rawski and Conroy, 2020).

Despite the contributions and implications of the research, there are potential limitations. First, The Feelings Thermometer is an effective tool for measuring explicit biases; however, other researchers have also shown that people can express explicit biases in more complex, multidimensional ways (Swim et al., 1995; Morrison et al., 2019; Dell’Armo and Tassé, 2021). It is possible that the pattern of explicit biases would differ employing these questionnaires. Of course, doing so would also preclude use of the Project Implicit dataset in most cases. Second, our study focused on managers in the US; thus, generalization of our findings is potentially limited to the US context. We do note that biases and mistreatment are patterns that exist around the world (Charlesworth et al., 2022), and thus, it is possible that the findings we observed in the US are applicable elsewhere, too. Future researchers should examine this possibility. Third, it is possible that people completed the IAT and other questionnaires more than once, and given that the data are anonymized, there is no way to account for this possibility. In addition, the same demographic information was not consistently available across all datasets, so we only analyzed the participants’ race and gender and controlled for them, accordingly. We recognize, though, that other demographic characteristics might influence the results. Finally, the occupational data are coded based on the respondent’s answers; thus, a healthcare worker who is also a manager could indicate that that work in healthcare or a management occupation, but not both. This possibility noted, respondents are likely to provide the occupation most relevant to them.

In addition to the aforementioned areas of future research, other inquiries are warranted. Specifically, people in community and social services; educational instruction and library; and arts, design entertainment, sports, and media consistently reported less bias than people working in management. What characteristics of people in the former occupations contribute to these differences? Are variations in the educational preparation, nature of the work, or other characteristics? If there are differences, to what extent are they transferable to people in other occupations, such as those working in management. Given the need to reduce biases among people working in management, such understandings are needed.

## Data availability statement

Publicly available datasets were analyzed in this study. The data were available from the Center for Open Science (https://osf.io/y9hiq/).

## Author contributions

GC developed the idea, collected the data, analyzed the data, and wrote the manuscript. HC collected the data, cleaned the data, and helped analyze the data. Both authors contributed to the article and approved the submitted version.

## Footnotes

1
https://implicit.harvard.edu/implicit

2
https://osf.io/y9hiq/#!

3
https://www.bls.gov/soc/2018/major_groups.htm
